# Malaria Policy Advisory Committee to the WHO: conclusions and recommendations of eighth biannual meeting (September 2015)

**DOI:** 10.1186/s12936-016-1169-x

**Published:** 2016-02-24

**Authors:** WHO Malaria Policy Advisory Committee and Secretariat

**Affiliations:** Global Malaria Programme, World Health Organization, 20 Avenue Appia, 1211 Geneva 27, Switzerland

**Keywords:** WHO, Malaria, Policy making, Mosquito control, Drug resistance, Surveillance, Elimination, *Plasmodium falciparum*, *Plasmodium vivax*

## Abstract

The Malaria Policy Advisory Committee (MPAC) to the World Health Organization held its eighth meeting in Geneva, Switzerland from 16 to 18 September 2015. This article provides a summary of the discussions, conclusions and meeting recommendations. Meeting sessions included: recommendations from the Evidence Review Group (ERG) on mass drug administration; recommendations from the ERG on malaria in pregnancy; recommendations on when to scale back vector control; feedback on the *Plasmodium vivax* technical brief and the recommendation for G6PD testing before treatment; updates on artemisinin and artemisinin-based combination therapy resistance and the Greater Mekong Subregion elimination strategy; an update from the working group on malaria terminology; and updates on malaria elimination in the World Health Organization European region, the ERG on malaria elimination, and World Health Organization reform to support innovation, efficiency and quality in vector control tools. Policy statements, position statements, and guidelines that arise from the MPAC meeting conclusions and recommendations will be issued formally and disseminated to World Health Organization Member States by the World Health Organization Global Malaria Programme.

## Background

The Malaria Policy Advisory Committee (MPAC) to the World Health Organization (WHO) held its eighth biannual meeting from 16 to 18 Sep 2015 in Geneva, Switzerland, following its meetings in February and September 2012, March and September 2013, March and September 2014, and March 2015 [1–7]. This article provides a summary of the discussions, conclusions and recommendations from the September 2015 meeting^1^ as part of the *Malaria Journal* thematic series “WHO global malaria recommendations” [8].

The following sections of this article provide details and references for the meeting sessions on: mass drug administration; malaria in pregnancy; when to scale back vector control; the *Plasmodium vivax* technical brief and the recommendation for G6PD testing before treatment; artemisinin and artemisinin-based combination therapy (ACT) resistance and the Greater Mekong Subregion elimination strategy; malaria terminology; malaria elimination; and WHO reform to support innovation, efficiency and quality in vector control tools.

The MPAC discussion and recommendations related to these topics, which took place partially in closed session, are also included. MPAC decisions are reached by consensus [9]. The next meeting of the MPAC will be held on 16–18 Mar 2016 [10].

### Report from the WHO Global Malaria Programme

Following a welcome by the Chair of MPAC, the Director of the WHO Global Malaria Programme (WHO-GMP) provided MPAC members with an update on WHO-GMP’s activities since their last meeting [11]. Most importantly, the WHO *Global Technical Strategy for Malaria* (*2016*–*2030*) was endorsed by the World Health Assembly in Geneva in May 2015 [12, 13] and, together with the companion document *Action and Investment to defeat Malaria* (*2016*–*2030*) [14] developed by the Roll Back Malaria (RBM) partnership, was jointly launched at the third International Conference on Financing for Development in Addis Ababa in July 2015. Regional implementation plans are currently under development and, with the exception of the European region, all regional consultations will take place by the end of 2015.

The Director’s report included an overview of anticipated guidance due in 2016 as well as guidance published on the WHO-GMP website since the last MPAC meeting in March. Guidance published in 2015 included the third edition of the WHO *Guidelines for the treatment of malaria* [15], the second edition of the operational manual for indoor residual spraying (IRS) for malaria transmission, control and elimination [16], a case study of the successful elimination and prevention of re-establishment of malaria in Tunisia [17], and the technical brief on the control and elimination of *P. vivax* malaria [18].

The Director provided an update on the new WHO-GMP departmental structure and its strategic priorities which are in line with the goals and targets of the new WHO *Global Technical Strategy for Malaria (2016*–*2030).* The new matrix structure includes the four existing but re-named units—drug efficacy and response; prevention, diagnostics and treatment; surveillance, monitoring and evaluation; and, entomology and vector control—together with three new cross-cutting units—strategy, evidence and economics; elimination; and, technical support and capacity building. Two team lead positions are currently under recruitment: Elimination, and Surveillance, Monitoring and Evaluation.

Updates from the WHO Regional Offices highlighted the key message of a global malaria situation where there has been both progress and challenges. For example, in the Americas region, with the exception of Haiti and Venezuela, all countries showed an overall 50–100 % decrease in malaria morbidity since 2000. However, some of these gains were unstable, with substantial yearly fluctuations. A similar scenario was present in the Eastern Mediterranean region; the European region has reported no cases since 2014. In the African region, even though malaria incidence decreased by 34 % and malaria mortality declined by 54 % from 2000 to 2013, huge gaps in intervention coverage still remain. For example, in 2013, only 29 % of households had enough insecticide-treated mosquito nets (ITNs) for all household members. Covering all populations at risk for malaria, especially mobile or migrant populations, is a particular challenge in the South-East Asian and Western Pacific regions as well.

The WHO-GMP Director provided updates to MPAC members on the changes that are being implemented across the WHO-GMP’s Technical Expert Groups (TEGs), in order to align them with the department’s new organizational structure. There will be five TEGs in all: chemotherapy; antimalarial drug efficacy and Response; vector control; surveillance, monitoring and evaluation; and financing, coverage and impact. Their terms of reference and membership are currently being reviewed. The Director also announced a new effort, to be launched soon and conducted over the next few years, to define and understand the implications of various determinants (including those beyond health, such as climate change) on the potential for malaria eradication. More details on this study group will be announced shortly.

### Recommendations from the Evidence Review Group on mass drug administration (MDA)

Mass drug administration (MDA) has received renewed interest from countries and funders over the past decade in the context of malaria elimination, as part of multidrug resistance containment, and more recently in emergency situations such as the West African Ebola outbreak. To assist in updating WHO recommendations developed in 2010, WHO-GMP convened an Evidence Review Group (ERG). This ERG met from 20 to 22 Apr 2015 to review recent published and unpublished evidence on the use of MDA, mass screening and treatment (MSAT) and focal screening and treatment (FSAT) in specific epidemiological settings.

The specific objectives of the ERG were to:

1. Review all available published and unpublished reports on the impact of MDA, MSAT and FSAT on malaria transmission, building on the recent Cochrane review, and a recent qualitative review.
2. Review the results of experiences and unpublished studies of large-scale implementation of MDA in Comoros, Sierra Leone, the Myanmar-Thai border, Vanuatu and Viet Nam and of MSAT and FSAT in Cambodia, Kenya, Zambia and Zanzibar.
3. Evaluate the role of the concomitant administration of single low-dose primaquine (PQ) (0.25 mg base/kg) as a *Plasmodium falciparum* gametocytocide, together with the artemisinin-based combination therapy (ACT) deployed for MDA.
4. Define the specific conditions for application of MDA, MSAT and FSAT to reduce malaria transmission in terms of endemicity, medicines and dosages, use of diagnostics, timings and number of MDA rounds, concomitant implementation of vector-control measures, and optimum strategies to ensure community uptake and pharmacovigilance.
5. Identify research gaps and provide recommendations on data requirements, study methods and ethical considerations for research groups and policy-makers interested in further evaluating the role of MDA, MSAT and FSAT in reducing malaria transmission.

The full ERG meeting report [19] and supporting background documentation [20] are available on the WHO-GMP website.

MPAC members thanked the ERG for the thoroughness of their evidence review, and for the subsequent work on grading of the evidence [21], modelling [22] and costing [23] of MDA programmes, which was also presented. General points raised during the MPAC discussion included: the need for a clear objective for MDA and a clear definition of the target population, the method and duration of delivery, and post-MDA activities; that MDA should be combined with other malaria control activities to sustain the gains, particularly in reducing the vectorial capacity, and to make use of existing delivery systems where possible; and that community engagement is essential for successful MDA campaigns. Discussion based on the modelling data stressed the importance of effective coverage, which is likely to be more important than the number and timing of rounds. In addition, MDA appeared to be more effective in low than high transmission situations, but optimum timing was dependent upon the objective of the MDA, for example, interruption of transmission versus morbidity reduction.

MPAC concluded that although there is generally weak evidence on which to base recommendations, there is a need to provide the global malaria community with some guidance. As a result, MDA is recommended only in the very specific circumstances outlined below.

Following MPAC input during the closed session of the meeting, WHO-GMP has published the following recommendations, which are available in full on their website [24]:

1. Use of MDA for the elimination of *P. falciparum* malaria can be considered in areas approaching interruption of transmission where there is good access to treatment, effective implementation of vector control and surveillance, and a minimal risk of re-introduction of infection.
2. Given the threat of multidrug resistance and the WHO call for malaria elimination in the Greater Mekong Subregion (GMS), MDA may be considered as a component of accelerated malaria elimination efforts in areas of the GMS with good access to treatment, vector control and surveillance.
3. Use of time-limited MDA to rapidly reduce malaria morbidity and mortality may be considered for epidemic control as part of the initial response, along with the urgent introduction of other interventions.
4. Use of time-limited MDA to reduce malaria morbidity and mortality may be considered during exceptional circumstances when the health system is overwhelmed and unable to serve the affected communities.
5. In the absence of sufficient evidence, WHO does not recommend the use of MDA in situations other than for areas approaching elimination, epidemics, and complex emergencies, as specified above (see 1–4).
6. Mass primaquine prophylactic treatment, requiring pre-seasonal MDA with daily administration of primaquine for two weeks without G6PD testing, is not recommended for the interruption of *P. vivax* transmission.
7. Mass screening and treatment and focal screening and treatment for malaria are not recommended as interventions to interrupt malaria transmission.
8. Medicines used for MDA must be of proven efficacy in the implementation area and preferably have a long half-life. WHO recommends that a medicine different from that used for first line treatment be used for MDA. Programmes should include monitoring of efficacy, safety and the potential emergence of resistance to the antimalarial medicines deployed for MDA.
9. WHO supports the need for more research on the optimum methods of implementing MDA programmes, promoting community participation and compliance with treatment, and evaluating their effectiveness. Modelling can help guide the optimum method of administering MDA in different epidemiological circumstances and predict its likely impact.

### Recommendations from the Evidence Review Group on malaria in pregnancy

Because malaria in pregnancy (MiP) is a major, preventable cause of maternal morbidity and poor birth outcomes, WHO recommends the use of ITNs, effective case management of malaria and anaemia in pregnant women, and in areas of moderate to high malaria transmission in sub-Saharan Africa, intermittent preventive treatment in pregnancy (IPTp) with sulfadoxine-pyrimethamine (SP). In recent years, an alternative preventive strategy—intermittent screening and treatment in pregnancy (ISTp) using rapid diagnostic tests (RDTs) and ACT during antenatal care (ANC) visits—has been evaluated in several countries. Moreover, multiple recent studies have assessed the safety of using ACTs in the first trimester of pregnancy. WHO convened an ERG in Geneva from 13 to 16 Jul 2015 to review this new evidence and develop recommendations on the efficacy and cost-effectiveness of (ISTp) compared to IPTp-SP for the prevention of MiP, and on the safety of ACTs for malaria treatment in the first trimester of pregnancy.

The objectives of the ERG were to:

(a) Compare ISTp with IPTp-SP, specifically:

1. Review all available published and unpublished reports on the efficacy and safety of ISTp compared to IPTp for prevention of the adverse consequences of MiP.
2. Review all available reports on the acceptability of ISTp under trial conditions.
3. Review results of cost-effectiveness analyses (CEA) of ISTp.
4. Review the recent evidence on the effect of sub-microscopic infections on maternal and infant outcomes.
5. Review available published and unpublished reports on the impact of SP resistance on the effectiveness of IPTp-SP.
6. Review results of recently completed clinical trials evaluating the efficacy and safety of dihydroartemisinin-piperaquine (DHA-PPQ) for IPTp.
7. Based on the evidence reviewed, consider whether either ISTp or IPTp-DHA-PPQ could be recommended as a potential alternative to IPTp-SP in some areas with high SP resistance and/or very low transmission.

(b) Review the safety of ACT in early pregnancy, specifically:

1. Review the evidence of embryotoxicity of artemisinin derivatives from animal studies.
2. Review available published and unpublished reports on exposures to artemisinin derivatives in the first trimester of pregnancy compared to other anti-malarial medicines.
3. Review results of recent clinical trials evaluating the efficacy and safety of different artemisinin-based combinations for malaria treatment in the second and third trimester of pregnancy.
4. Based on the evidence reviewed, consider whether the current WHO recommendations on use of ACT in the first trimester of pregnancy could be updated.

The full meeting report [25] is available on the WHO-GMP website. In summary, the ERG concluded that ISTp should not be recommended as an alternative to IPTp-SP, which remains highly cost effective, and ACT compared to quinine appear to be comparably safe to use in the first trimester of pregnancy, although as with all drug exposures, there is a need for continued pharmacovigilance.

MPAC thanked the MiP ERG for their efforts. WHO-GMP will continue to promote the adoption and implementation of the updated IPTp-SP recommendation [26], and encouraged scaling-up coverage of three or more doses of IPTp-SP. In addition, MPAC recommended that new data on miscarriage and congenital malformations following exposure to artemisinin derivatives in the first trimester of pregnancy should be included in the update of the WHO *Guidelines for the treatment of malaria* [15], and follow the process established by WHO so that ACTs may be considered for inclusion as a first-line therapeutic option.

While WHO-GMP works to formally update the online version of the guidelines through the appropriate mechanisms, MPAC’s recommendations [27] are listed below:

1. Recent comparative studies have shown that intermittent screening and treatment in pregnancy (ISTp) with RDTs and ACT resulted in a higher proportion of maternal infections and clinical malaria during pregnancy compared to intermittent preventive treatment in pregnancy (IPTp) with SP given during ANC visits. The effects of ISTp on birth weight varied. In some studies, ISTp with artemether-lumefantrine was not inferior to IPTp in preventing low birth weight. In other studies, ISTp with dihydroartemisinin-piperaquine (DHA-PPQ) resulted in a lower mean birth weight compared with IPTp-SP in paucigravidae in areas of high malaria transmission and high SP resistance. ISTp is also less cost-effective than IPTp-SP and, for these reasons, it is not recommended as an alternative to IPTp-SP.
2. IPTp-SP remains highly cost-effective in preventing the adverse consequences of malaria on maternal and fetal outcomes, and should, therefore, be actively scaled up in line with the current WHO recommendations. IPTp-SP also remains effective in areas where quintuple-mutant haplotypes of *P. falciparum* to SP are highly prevalent. Further research on the relationship of SP resistance markers and IPTp effectiveness should be done, particularly in areas where transmission and thus maternal immunity have declined substantially in recent years.
3. The threshold level of malaria transmission below which IPTp-SP is no longer cost-effective has not been identified. Therefore, in areas where IPTp-SP is implemented and transmission has been reduced to low levels as a result of successful control strategies, WHO recommends continued IPTp-SP implementation until the area approaches interruption of transmission.
4. An association between sextuple mutant haplotypes of *P. falciparum* and decreased birth weight has been reported in observational studies in a few sites in East Africa. Further studies are required to assess this and to devise the best and most cost-effective prevention strategies in areas of very high SP resistance. One potential strategy to be tested is to provide a single RDT screening and ACT treatment at the first ANC visit during the second trimester, in addition to the continued delivery of IPTp-SP.
5. Recent studies have shown that IPTp with DHA-PPQ does not reduce the incidence of low birth weight compared to IPTp-SP, but that it is more efficacious in reducing maternal malaria parasitaemia and anaemia at delivery, incidence of malaria infection and clinical malaria during pregnancy, and stillbirths and early infant mortality (i.e., within 6–8 weeks). More research is needed to evaluate the impact of DHA-PPQ for IPTp in preventing low birth weight, safety of repeated doses, and adherence to the required 3-day regimen.
6. New evidence from 1025 pregnancies with confirmed artemisinin exposure in the first trimester in South-East Asia and sub-Saharan Africa indicates that artemisinins are not associated with an increased risk of miscarriage, stillbirths or major congenital malformations compared to non-artemisinin regimens. Moreover, comparison of carefully documented and prospectively collected safety data on women exposed only to artemisinin-based treatment with data collected on women exposed only to quinine in the first trimester of pregnancy showed that artemisinin was associated with a significantly reduced rate of miscarriage compared to quinine. MPAC recommends the review of the WHO *Guidelines for the treatment of malaria* to consider the timely inclusion of ACT as a first-line therapeutic option for uncomplicated falciparum malaria.

### Recommendations on when to scale back vector control

Members of the Vector Control Technical Expert Group (VC TEG) and the WHO-GMP Secretariat presented their conclusions from a comprehensive literature review and mathematical simulation model on when to scale back vector control, a core component of malaria prevention, in areas where malaria transmission has been reduced [28]. WHO currently recommends universal coverage with effective vector control for all persons at risk of malaria. Universal coverage is defined as one ITN for every two persons at risk of malaria, and the population at risk (defined periodically on a sub-national level) includes all persons in geographical areas or localities with ongoing malaria transmission.

Since 2000, substantial expansion of funding has enabled significant scaling up of malaria prevention, diagnostic testing and treatment. However, given the general decline in malaria transmission in many settings, WHO Member States have recently requested guidance from WHO-GMP on the circumstances under which it may be appropriate to scale back vector control interventions to targeted deployment in specific geographic areas. This request has been prompted largely by the recognition that the epidemiology of malaria has been altered in some settings as a result of years of sustained, effective, malaria control. However, there is a concern that this may lead to a perception that the discontinuation of vector-control implementation in such settings will be associated with a minimal risk of resurgence, and that such scale-back is an appropriate way for malaria programmes to better allocate resources.

The modelled scenarios presented to MPAC examined the epidemiological implications of reducing the coverage of ITNs and IRS to no coverage under conditions of differing levels of: (a) baseline (i.e., pre-intervention) entomological inoculation rates (EIR); (b) infection importation rates; (c) disease surveillance coverage; and (d) case management coverage. The results, highlighted in greater detail in the meeting presentation itself and available online [29], indicated that the scale-back of malaria vector control was associated with a high probability of malaria resurgence, including most areas in which malaria transmission was very low or had been interrupted (i.e., no local transmission). Even in areas where there are substantial reductions in malaria transmission (indicated by an annual incidence of <1 local case per 1000 population), discontinuing vector control conferred a high risk of malaria resurgence in most situations, This risk increased in contexts of relatively high receptivity (defined as the ability of an ecosystem to allow transmission of malaria), high rates of vulnerability (defined as the frequency of influx of infected individuals or groups and/or infective anophelines), and low coverage of disease surveillance and case management.

The analysis found that the situations with a high probability of resurgence were likely to correspond most closely with malaria-endemic areas of sub-Saharan Africa. The probability of resurgence was low only in scenarios with low historic EIRs, low infection importation rates, and high coverage of both disease surveillance and case management. Such scenarios are found mainly in countries outside of sub-Saharan Africa that are currently experiencing very low malaria incidence. The precise measures of malaria receptivity, vulnerability, and the levels of these parameters at which scale-back of vector control caries minimal risk of resurgence, still remain to be comprehensively defined. Similarly, it is difficult to predict whether zero local transmission can be maintained in the absence of vector control. Moreover, where there has been minimal change in inherent malaria transmission potential, the stability of the malaria parasite–vector relationship following interruption of malaria is not well understood. Further evaluations of the specific criteria for identifying areas where vector-control scale-back would carry a low risk of malaria resurgence are, therefore, required before further conclusions can be drawn.

MPAC made suggestions to better refine definitions for receptivity and vulnerability so that they matched updated malaria terminology (these have been reflected in the summary above), and to clarify a few points in the main text. The wording of the recommendations emphasizes the definition of what an “area” is (i.e., it is to be based on the availability of reliable disaggregated disease surveillance data and feasibility for decisions on vector control implementation—and not necessarily on administrative boundaries). This embraces the concept of geographical targeting of vector control, including investments in entomological monitoring as part of any scale-back. Because the recommendations build on current policy—WHO continues to recommend effective vector control in areas where there continues to be malaria transmission while acknowledging that new vector control tools are urgently needed—they are published in the form of an information note to assist countries, and their funders, to translate these in the planning and implementation for malaria control programmes [30].

The information note contains the following recommendations:

1. In areas with ongoing local malaria transmission (irrespective of both the pre-intervention and the current level of transmission), the scale-back of vector control is not recommended. Universal coverage with effective malaria vector control (including the use of new vector control tools when they become available) of all persons in such areas should be pursued and maintained.
2. In areas where transmission has been interrupted, the scale-back of vector control should be based on a detailed analysis that includes assessment of the receptivity and vulnerability, active disease surveillance system, and capacity for case management and vector control response.
3. Countries and partners should invest in health systems particularly in the strengthening of disease and entomological surveillance, as identification of areas for geographical scale-back as well as timely detection and appropriate response to resurgence depend on this capacity.

MPAC members underscored the critical need for all countries with ongoing malaria transmission, and in particular those approaching elimination, to build and maintain strong capacity in disease and entomological monitoring in order to provide useful setting-specific information on which to base decisions, including the ability to respond to possible resurgences. For example, in areas where transmission has been reduced significantly, active case detection will be needed because at this stage every case matters and so should be found, treated, and reported. Such capacity will be a pre-condition for evaluating the potential for geographical scale-back of vector control.

### Feedback on the section of the *Plasmodium vivax* technical brief related to recommendation for G6PD testing before radical cure with primaquine

The WHO *Guidelines for the treatment of malaria* [15] contain recommendations for the treatment of *P. vivax* and *Plasmodiumovale* that are based on the need to radically cure patients using primaquine (the only available anti-relapse medicine) while at the same time minimizing the risk of primaquine-induced acute haemolysis in those who are deficient in the enzyme glucose-6-phospate dehydrogenase (G6PD). The guidelines recommend that patients with confirmed *P. vivax* or *P.ovale* malaria who are not aware of their G6PD status should be tested before the administration of radical cure with primaquine. The guidelines provide recommendations for primaquine anti-relapse therapy in both G6PD normal and G6PD deficient patients. In addition, the guidelines also specify that when G6PD testing is not available, a decision to administer or withhold primaquine may still have to be based on weighing the benefits of radical cure against the haemolytic risk posed by primaquine.

These recommendations on the radical cure of *P.vivax* infections are also reiterated in *Control and elimination of Plasmodium vivax malaria—A technical brief* [18], a WHO-GMP publication that deals exclusively with the control and elimination of *P.vivax* malaria. This technical brief was launched on 29 Jul 2015 at a global meeting held in New Delhi that was attended by countries in all WHO Regions with endemic *P. vivax* malaria. The launch was followed by a 2-day meeting in which participating countries deliberated on the translation of the guidelines into policy and strategy for their national malaria control programmes (NMCPs). The meeting brought to light two main issues that will make implementing the WHO recommendations challenging. They are:

- The limited availability of a robust, easy-to-use, point-of-care G6PD test restricts the ability to deploy primaquine for radical cure at the primary health care level. Promoting referral to higher level facilities where primaquine can be administered safely and G6PD testing performed would therefore need to be more explicit in the current recommendations so as not to compromise the schizonticidal treatment for *P. vivax* that is ongoing at the primary level. At present only early treatment of blood-stage infection can be accessed in peripheral health care settings, including at the community level.
- Some countries, particularly (but not only) in the Region of the Americas, are currently implementing radical cure for all patients at health facility level without testing for G6PD. The rationale for this approach is that the G6PD deficiency allele frequency is low in these areas, and therefore the benefits of providing primaquine radical cure for all *P.vivax* patients exceed the risk of primaquine-induced haemolysis. In these settings, full compliance with the new recommendation of testing before treatment could affect progress in the control of *P. vivax* malaria and potentially reverse the progress made.

The main conclusion from the Delhi meeting, which were summarized and presented to MPAC [31], were that there is a need for additional practical guidance from WHO-GMP to countries on:

- How NMCPs could progressively introduce quality G6PD tests that are currently available for all confirmed *P.vivax* patients before providing them with primaquine radical cure without compromising the existing and ongoing programmes aimed at achieving greater coverage for *P. vivax* treatment in general. Learning from early deployment can help when expanding the introduction of G6PD testing to areas where it is currently not deployed.
- How to perform a risk–benefit analysis at national level on administering primaquine radical cure when a patient’s G6PD status is unknown, considering the prevalence and type of G6PD deficiency in the country, the frequency and risk of *P. vivax* relapses, the availability of point of care G6PD tests, the capacity to interpret those tests correctly, and the capacity of the health care system to detect and manage the risk of primaquine-induced haemolysis.

MPAC members agreed with the feedback provided by *P. vivax* endemic countries and supported their request for additional guidance from WHO-GMP. Their advice to WHO-GMP was for the current WHO recommendations to remain unchanged in the WHO *Guidelines for the treatment of malaria*, which should serve as a primary source document, and that the *P. vivax* technical brief and any other similar subsequent documents always refer to the WHO *Guidelines for the treatment of malaria* [15]. It was recommended that WHO-GMP produce additional implementation guidance to assist countries with the practicalities of G6PG testing.

### Update on artemisinin and ACT resistance with special focus on the Greater Mekong Subregion (GMS), including the GMS elimination strategy

WHO-GMP updated MPAC members on the current status of artemisinin and ACT resistance, which is available on the WHO-GMP website [32]. Of particular interest was the response to artemisinin resistance and move towards the goal of malaria elimination in the GMS, where the incidence of malaria has been greatly reduced over the past 10–20 years. However, this has been coupled with concern that in certain areas within the GMS, *P. falciparum* is becoming increasingly resistant to anti-malarial medicines. The current situation is particularly worrisome at the border between Cambodia and Thailand, where *P. falciparum* could become untreatable within a few years. In addition, molecular studies have confirmed that artemisinin resistance has emerged independently in multiple areas of the GMS. In response, MPAC recommended at its September 2014 meeting the adoption of the goal of elimination of *P. falciparum* in the GMS by 2030. Subsequently, at the World Health Assembly in May 2015, WHO-GMP launched a *Strategy for malaria elimination in the Greater Mekong subregion (2015*–*2030)* [33], which was endorsed by all the GMS countries (Cambodia, Lao People’s Democratic Republic, Myanmar, Thailand, Viet Nam). An update on progress of the strategy implementation was given [34].

Some of the highlights from GMS country updates [35] on ACT efficacy were:

- Cambodia: a consensus meeting on the national treatment policy for *P. falciparum* was held in January 2014. As a result, artesunate-mefloquine (ASMQ) has been re-introduced as first-line treatment, replacing dihydroartemisinin-piperaquine (DHA-PPQ), since the proportion of *P. falciparum* strains with multiple *pfmdr1* copy numbers (which confer mefloquine resistance) is currently minimal in the area.
- Lao People’s Democratic Republic: the emergence of artemisinin resistance in southern Lao PDR is supported by the identification in 2013 of the presence of *k13* mutants (mainly C580Y and R539T) in the circulating parasite populations. However, the therapeutic efficacy of artemether-lumefantrine (AL) has not been affected, and cure rates have remained high since 2005. Containment activities started in 2014, and therapeutic efficacy studies (TES) are now being conducted in Attapeu, Champasak and Sekong provinces.
- Myanmar: studies evaluating the presence of *k13* mutants have shown that the predominant *k13* mutant found in Myanmar is likely to have arisen independently rather than to have spread from Cambodia. A new *k13* propeller polymorphism (F446I) associated with delayed parasite clearance was detected as early as 2013 along both the China–Myanmar border and the India–Myanmar border. Research is ongoing to validate the role of this new mutant in artemisinin resistance but preliminary results suggest that the F446I mutation is associated with a lower level of artemisinin resistance compared to C580Y. However, despite a high prevalence of the *k13* F446I in Myanmar, ACT efficacy remains high on both sides of the border between India and Myanmar.
- Thailand: during a consensus meeting held in 2015, DHA-PPQ became the first-line treatment in the country, and its efficacy is currently being evaluated.
- Viet Nam: TES conducted since 2010 using DHA-PPQ reported a treatment efficacy of more than 95 %, despite a day-3 positivity rate of up to 36 %.

WHO-GMP stressed that despite the delayed parasite clearance associated with artemisinin resistance in some areas of the GMS, ACT still remains the most effective treatment for uncomplicated *P. falciparum* malaria. Most patients with delayed parasite clearance are cured, as long as the partner drug remains effective. It is imperative that routine monitoring of therapeutic efficacy continues to ensure that the recommended ACT is effective, that changes in national treatment policies can be implemented in a timely manner, and that artemisinin resistance can be detected early. The assessment of K-13 propeller-region mutants will greatly facilitate the tracking of artemisinin resistance as it emerges.

Given the commitment to eliminate *P. falciparum* malaria in the GMS set out in the recently launched *Strategy for malaria elimination in the Greater Mekong Subregion (2015*–*2030)* [33], MPAC was provided with an update from the Coordinator of the emergency response to artemisinin resistance (ERAR) and Mekong Malaria Elimination Hub [36]. The update outlined the goals, objectives, milestones, targets and main interventions of the strategy, including progress with the roll out of the strategy. Because the strategy was only launched in May, the development and adaptation of national malaria elimination strategies to fall in line with the overall strategy is ongoing. Ongoing activities include setting up the appropriate regional governance structures, conducting training, and surveillance, monitoring and evaluation. MPAC members were pleased to see the improvement in surveillance in the region, including the collection of the baseline information needed for the GMS elimination strategy. However, in future they requested that WHO-GMP schedules more time for GMS elimination strategy updates, which will feature more detailed and up-to-date data on progress and challenges.

### Malaria terminology

MPAC members welcomed the WHO-GMP initiative to update the WHO publication, *Terminology of malaria and of malaria eradication*, which dates back to 1963. Several WHO publications over the past 10 years have included a glossary of terms related to malaria prevention, control, elimination and surveillance. However, the terminology of malaria has not been comprehensively reviewed for 50 years and is in need of an update, to archive terms no longer in use, and to clarify terms so that they can be consistent in meaning across documents.

WHO-GMP has taken a phased approach in updating malaria terminology, further details of which are available on the WHO-GMP website as part of the MPAC meeting background documents [37, 38]. The first stage of the process was a desk review that focused on terms having programmatic relevance, being related to malaria elimination and eradication, and having conflicting definitions and use. This process was carried out between April and May 2015, and resulted in a total of 292 terms identified with draft definitions proposed, in some cases with an explanatory note. Terms were divided into four groups related to elimination, vector control, surveillance, and diagnosis and treatment, with many terms relevant to both surveillance and elimination.

These terms and their definitions were then submitted to the members of the WHO Drafting Committee on Malaria Terminology who were asked to classify them into three groups: (a) terms that were and are still relevant and properly described; (b) terms that have been used in the past and have value for historical purposes, but are no longer in current use; and (c) terms that are relevant today but may have taken on a new meaning and different use. After an initial review, the committee was convened for a consultation in Geneva on 2–3 Jun 2015, to refine all definitions. A concerted effort was made to simplify definitions as much as possible, and, as a result, the recommended definitions tended to be short with an explanatory note to provide qualifying information. Following extensive work on the definitions, the drafting committee considered 153 terms as being properly described, 38 were proposed for archiving, and 101 terms were identified as requiring additional inputs.

In order to collect additional inputs on these 101 terms in a systematic way, WHO-GMP developed an online survey and issued a weblink with passcodes that were sent to 30 identified institutions or groups. The survey was carried out between 6 and 26 Jul 2015, and responses were obtained from the majority of institutions on most of the survey categories. All inputs were reviewed and compiled by the WHO-GMP Secretariat and the suggested modifications then submitted to the WHO Drafting Committee for review by email exchanges. The consolidated result of this work in the form of a glossary [39] was submitted to MPAC for final review, together with specific consideration on the term “malaria case” [40], which generated significant debate among the members of the Drafting Committee and external reviewers.

MPAC members commended the Drafting Committee and WHO-GMP on the rigorous review of malaria terminology that had been conducted in a relatively short period of time. MPAC members were asked to advise on three issues: advice on malaria case definition [40]; feedback on the glossary with proposed terms and definitions [39]; and advice on the process for reviewing and incorporating new terms. The definition of a “malaria case” was debated extensively by MPAC and other attendees. The majority felt that there should be one definition related to the presence of malaria infection with two possible application groups: (1) individuals who present with clinical signs and symptoms, and (2) those with asymptomatic infection. The Drafting Committee provided specific advice on the definition of “malaria case” with multiple applications in surveillance and for directing malaria control and elimination efforts. Following the contribution by MPAC the definition will be updated and a new proposed text will be shared with MPAC members before it is finalized.

All present were encouraged to submit suggested edits to the glossary to the Drafting Committee via WHO-GMP. The full glossary of updated terminology will be made available for dissemination via the WHO-GMP website. For future modifications to the glossary, MPAC members suggested that each of the respective TEGs should review terminology in their subject areas as an ongoing process. The proposed new terms would then be shared via the WHO-GMP Secretariat and reviewed by a standing WHO Drafting Committee on Malaria Terminology. This Committee would then propose final wording, after which WHO-GMP would add the new terms to an on-line glossary on its website. The online glossary would be promoted through scientific journals, and with members of the global malaria community, to adhere to the updated definitions once finalized.

### Updates on malaria elimination in the WHO European region, the Evidence Review Group on malaria elimination, and WHO reform to support innovation, efficiency and quality in vector control tools

The final open session of the MPAC meeting featured several brief updates for members that were primarily for information purposes. These are summarized in brief in this section of the report, although the full presentations are available along with all other meeting documents online [41].

Firstly, an update that drew congratulatory remarks from MPAC and other meeting observers, was the news that in 2015 there have been no indigenous cases of malaria reported in the WHO European region [42]. Despite there being an upsurge in cases in the 1990s in many countries in the region following the breakup of the Soviet Union and a lack of resources for control efforts, malaria has been brought under control through the reinvigoration of malaria control programmes and provision of sufficient financial resources to support their work. There remains a risk of epidemics in countries with large influxes of refugees, such as Turkey, and a risk of imported cases from Afghanistan into Tajikistan, but certified elimination within the WHO European region is now within reach. MPAC commended the countries and the Regional Office on their hard work and noted that there were several lessons to be learned about the journey to malaria elimination from the Region’s experience.

The second update concerned malaria elimination and the process for certification [43]. WHO-GMP, via an ERG, is currently updating its manual of guidelines to countries on the elimination process and its certification. The new manual will be a major revision of the current guidelines since the malaria landscape has changed dramatically since the first elimination guidance manual was published in 2007 [44]. The updated manual will also be aligned with the recently launched WHO *Global Technical Strategy for Malaria (2016*–*2030).* The process of updating the elimination manual is progressing well. The ERG met for the first time in July 2015, and will meet again two more times before the draft manual is reviewed by MPAC at its meeting in September 2016. It is based on the scenario that all malaria-endemic countries can accelerate efforts towards elimination through combinations of interventions that are tailored to local contexts, without the restrictions of the phased elimination approach currently in use.

In terms of the certification process for elimination, MPAC endorsed a plan for a revised and more streamlined process to include an increased role for national committees as well as MPAC, in collaboration with dedicated team of observers/certifiers who will conduct country visits. The new certification process will be outlined in more detail in the updated elimination manual.

Finally, MPAC members received an update on WHO reform of activities which support innovation in vector control [45, 46], which is part of a broader process in which WHO is one partner. WHO reported that reform is underway to improve innovation, to streamline current vector control advisory committees at WHO, and make more transparent the process for bringing new, effective, and high-quality vector control products to market. The new review committee structure within WHO is still being finalized and the transition process will take some time, but MPAC members welcomed the change because of the benefits it will bring to vector-control product manufacturers, national regulatory authorities, the procurement sector, and most importantly, WHO Member States and their national malaria control programmes.

## Discussion

The wording for recommendations was finalized by MPAC during their closed session and, in some cases, via email following the meeting; conclusions have been included in the summaries of the meeting sessions above, and links to the full set of meeting documents from the open sessions are provided as references.

Policy recommendations in line with MPAC suggestions will be issued formally and disseminated to WHO Member States by WHO-GMP and the WHO Regional Offices. Conclusions and recommendations from MPAC meetings are published in the *Malaria Journal* as part of this series.

On-going engagement with and attendance by interested stakeholders at MPAC meetings continues to be strong, although it was noted that more can be done to publicize open registration, especially to encourage attendance by research and development organizations who might not otherwise be aware that their presence as observers, as with all stakeholders who attend MPAC meetings as observers, is most welcome.

## Conclusion

WHO-GMP thanked those MPAC members (Salim Abdulla, Elfatih Malik, Patricia Graves, and Allan Schapira) who will conclude their service on the committee at the end of 2015, and welcomed the new members who will be replacing them starting in 2016—Ahmed Adeel, Tom Burkot, Gabriel Carrasquilla, Azra Ghani and Gao Qi.

The meeting feedback received from MPAC members, participants and observers [47] was generally positive. WHO-GMP and MPAC continue to welcome feedback, support, and suggestions for improvement of MPAC meetings from the global malaria community via the WHO-GMP website [9]. The next meeting of the MPAC will take place from 16 to 18 March 2016 in Geneva, Switzerland. Further information including the agenda and registration details will be made available in January 2016 on the MPAC page of the WHO-GMP website, although questions are welcome at any time [9].

## Abbreviations

ACT: artemisinin-based combination therapy
AL: artemether-lumefantrine
ANC: antenatal care
ASMQ: artesunate-mefloquine
DHA-PPQ: dihydroartemisinin-piperaquine
ERAR: WHO emergency response to artemisinin resistance
EIR: entomological inoculation rates
ERG: Evidence Review Group
FSAT: focal screening and treatment
G6PD: glucose-6-phosphate dehydrogenase
GMS: Greater Mekong Subregion
IPTp: intermittent preventive treatment of malaria in pregnancy
ISTp: intermittent screening and treatment of malaria in pregnancy
IRS: indoor residual spraying
ITN: insecticide-treated mosquito net
LBW: low birth weight
MDA: mass drug administration
MiP: malaria in pregnancy
MPAC: Malaria Policy Advisory Committee
NMCPs: national malaria control programmes
RDTs: rapid diagnostic tests
RBM: Roll Back Malaria
MSAT: mass screening and treatment
PQ: primaquine
SP: sulfadoxine-pyrimethamine
TEG: Technical Expert Group
WHO-GMP: World Health Organization Global Malaria Programme

## Footnotes

1 The complete set of all MPAC September 2015 meeting-related documents including background papers, presentations, and member declarations of interest can be found online at http://www.who.int/malaria/mpac/sep2015/en/.
